# Effectiveness of generic direct-acting agents for the treatment of hepatitis C: systematic review and meta-analysis

**DOI:** 10.2471/BLT.19.231522

**Published:** 2019-11-08

**Authors:** Hugo Perazzo, Rodolfo Castro, Paula M Luz, Mariana Banholi, Rafaela V Goldenzon, Sandra W Cardoso, Beatriz Grinsztejn, Valdilea G Veloso

**Affiliations:** a Instituto Nacional de Infectologia Evandro Chagas, Fundação Oswaldo Cruz, Avenida Brasil 4365, CEP 21040-360, Rio de Janeiro, Brazil.

## Abstract

**Objective:**

To compare the efficacy of generic direct-acting agents and brand-name medicines for treating hepatitis C virus (HCV) infection by conducting a systematic review and meta-analysis.

**Methods:**

We searched online databases for studies that reported sustained virological responses 12 weeks after the end of HCV treatment with generic direct-acting agents. We derived pooled proportions of treated patients with a sustained virological response from intention-to-treat and per-protocol analyses. In addition, we calculated the pooled relative risk (RR) of a sustained virological response brand-name versus generic direct-acting agents using a random-effects model (DerSimonian–Laird) from the data available. Between-study heterogeneity was assessed using the *I^2^* statistic.

**Findings:**

We identified 19 studies involving a total of 57 433 individuals from eight territories or regions. The pooled overall proportions of patients with a sustained virological response were 98% (95% confidence interval, CI: 97–99; 18 studies; *I^2^* = 94.1%) in per-protocol analyses and 96% (95% CI: 93–98; 8 studies; *I^2^* = 68.1%) in intention-to-treat analyses. The likelihood of a sustained virological response with brand-name medicines was similar to that with generic direct-acting agents (RR: 1.00; 95% CI: 0.98–1.02; *I^2^* = 0.0%). The likelihood of a sustained virological response was significantly higher in patients without than with cirrhosis (RR:1.03; 95% CI: 1.01–1.06; 7 studies) but was not significantly affected by either previous treatment (3 studies) or human immunodeficiency virus coinfection (3 studies).

**Conclusion:**

Generic direct-acting agents are highly effective for treating hepatitis C. Generic agents should be considered in resource-constrained settings for decreasing the burden of liver disease in HCV-infected patients.

## Résumé

**Objectif:**

Comparer l’efficacité des antiviraux à action directe génériques et des médicaments de marque pour traiter l'infection par le virus de l’hépatite C (VHC) à l’aide d’une revue systématique et d’une méta-analyse.

**Méthodes:**

Nous avons recherché dans les bases de données en ligne des études qui décrivaient une réponse virologique soutenue 12 semaines après la fin du traitement contre le VHC par antiviraux à action directe génériques. Nous avons obtenu les proportions combinées de patients traités présentant une réponse virologique soutenue à partir d’analyses en intention de traiter et per protocole. De plus, nous avons calculé le risque relatif (RR) combiné d’une réponse virologique soutenue avec les antiviraux à action directe de marque contre génériques à l’aide d'un modèle à effets aléatoires (DerSimonian–Laird) à partir des données disponibles. L’hétérogénéité entre les études a été évaluée en utilisant la statistique *I^2^*.

**Résultats:**

Nous avons identifié 19 études portant sur un total de 57 433 personnes réparties dans huit territoires ou régions. Les proportions totales combinées de patients présentant une réponse virologique soutenue étaient de 98% (intervalle de confiance, IC, à 95%: 97–99; 18 études; *I^2^* = 94,1%) dans les analyses per protocole et 96% (IC à 95%: 93–98; 8 études; *I^2^* = 68,1%) dans les analyses en intention de traiter. La probabilité d'une réponse virologique soutenue avec des médicaments de marque était similaire à celle obtenue avec des antiviraux à action directe génériques (RR: 1,00; IC à 95%: 0,98–1,02; *I^2^* = 0,0%). La probabilité d'une réponse virologique soutenue était sensiblement plus élevée chez les patients sans cirrhose que chez ceux ayant une cirrhose (RR: 1,03; IC à 95%: 1,01–1,06; 7 études), mais elle n’était pas significativement affectée par la prise d'un traitement antérieur (3 études) ou par une co-infection par le virus de l’immunodéficience humaine (3 études).

**Conclusion:**

Les antiviraux à action directe génériques sont très efficaces pour traiter l’hépatite C. Les médicaments génériques devraient être envisagés en cas de ressources limitées afin de réduire la charge des affections hépatiques chez les patients infectés par l’hépatite C.

## Resumen

**Objetivo:**

Comparar la eficacia de los antivirales genéricos de acción directa y los medicamentos de marca para el tratamiento de la infección por el virus de la hepatitis C (VHC) mediante la realización de una revisión sistemática y un metaanálisis.

**Métodos:**

Se realizaron búsquedas en las bases de datos en línea de estudios que notificaron respuestas virológicas sostenidas 12 semanas después del final del tratamiento contra el VHC con antivirales genéricos de acción directa. Se derivaron las proporciones agrupadas de los pacientes tratados con una respuesta virológica sostenida de los análisis por intención de tratar y por protocolo. Además, se calculó el riesgo relativo (RR) agrupado de un medicamento de marca de respuesta virológica sostenida versus antivirales genéricos de acción directa mediante un modelo de efectos aleatorios (DerSimonian–Laird) a partir de los datos disponibles. Se evaluó la heterogeneidad entre los estudios mediante la estadística *I^2^*.

**Resultados:**

Se identificaron 19 estudios con un total de 57.433 individuos de ocho territorios o regiones. Las proporciones generales agrupadas de pacientes con una respuesta virológica sostenida fueron del 98 % (intervalo de confianza del 95 %, IC: 97–99; 18 estudios; *I^2^* = 94,1 %) en los análisis por protocolo y del 96 % (IC del 95 %: 93–98; 8 estudios; *I^2^* = 68,1 %) en los análisis por intención de tratar. La probabilidad de una respuesta virológica sostenida con medicamentos de marca fue similar a la de los antivirales genéricos de acción directa (RR: 1,00; IC del 95 %: 0,98–1,02; *I^2^* = 0,0 %). La probabilidad de una respuesta virológica sostenida fue significativamente mayor en los pacientes sin cirrosis que con cirrosis (RR: 1,03; IC del 95 %: 1,01–1,06; 7 estudios), pero no se vio afectada significativamente por el tratamiento previo (3 estudios) o la coinfección por el virus de la inmunodeficiencia humana (3 estudios).

**Conclusión:**

Los antivirales genéricos de acción directa son altamente efectivos para el tratamiento de la hepatitis C. Los antivirales genéricos deben ser considerados en entornos con recursos limitados para disminuir la carga de la enfermedad hepática en pacientes infectados por el VHC.

## ملخص

**الغرض:**

مقارنة فعالية العوامل العامة ذات التأثير المباشر والأدوية ذات العلامات التجارية لعلاج عدوى فيروس التهاب الكبد الوبائي "سي" (HCV) عن طريق إجراء مراجعة منهجية وتحليل تلوي.

**الطريقة:**

لقد ****بحثنا في قواعد البيانات على الإنترنت عن دراسات أبلغت عن استجابات فيروسية مستدامة بعد 12 أسبوعًا من نهاية علاج التهاب الكبد الوبائي (HCV) باستخدام عوامل عامة ذات تأثير مباشر. لقد استخلصنا نسبًا مجمعة للمرضى المُعالّجين الذين أظهروا استجابة فيروسية مستدامة من تحليلات مقاصد العلاج والتحليلات لكل بروتوكول. بالإضافة إلى ذلك، قمنا بحساب المخاطر النسبية المجمعة (RR) الخاصة بالاستجابة الفيروسية المستدامة للعوامل ذات العلامة التجارية مقابل العوامل العامة ذات التأثير المباشر باستخدام نموذج للتأثيرات العشوائية (DerSimonian-Laird) من البيانات المتاحة. تم تقييم عدم التجانس بين الدراسات باستخدام الإحصاء .

**النتائج:**

حددنا 19 دراسة تتضمن 57433 فردًا إجمالًا من ثمانية أقاليم أو مقاطعات. وكانت النسب الإجمالية المجمعة للمرضى الذين يعانون من استجابة فيروسية مستدامة 98٪ (فاصل الثقة 95٪: 97 إلى 99؛ 18 دراسة؛ *I ^2 ^* = 94.1٪) في التحليلات لكل بروتوكول و96٪ (95٪ فاصل الثقة: 93 إلى 98؛ 8 دراسات؛ *I ^2 ^* = 68.1٪) في تحليلات نية العلاج. وتشابهت احتمالية حدوث استجابة فيروسية مستدامة مع الأدوية ذات العلامات التجارية لتلك التي تحدث مع العوامل العامة ذات التأثير المباشر (المخاطر النسبية المجمعة: 1.00؛ 95٪ فاصل الثقة: 0.98 إلى 1.02؛ *I ^2 ^* = 0.0٪). وكانت احتمالية حدوث استجابة فيروسية مستدامة أعلى بكثير في المرضى الذين يعانون من تليف الكبد (المخاطر النسبية المجمعة: 1.03؛ 95٪ فاصل الثقة: 1.01 إلى 1.06؛ 7 دراسات) ولكن لم يتأثر بشكل كبير إما بالمعالجة السابقة (3 دراسات) أو الإصابة بفيروس العوز المناعي البشري (3 دراسات).

**الاستنتاج:**

تُعتبر العوامل العامة ذات التأثير المباشر فعالة للغاية في علاج فيروس التهاب الكبد الوبائي "سي". يجب وضع العوامل العامة في الاعتبار في الأماكن محدودة الموارد، وذلك لتقليل عبء مرض الكبد في المرضى المصابين بعدوى فيروس التهاب الكبد الوبائي.

## 摘要

**目的:**

通过开展系统评审和元分析，比较通用直接药物和品牌药物治疗丙型肝炎病毒 (HCV) 感染的疗效。

**方法:**

我们搜索了在线数据库中报告的使用直接药物进行 HCV 治疗 12 周后的持续病毒性应答方面的研究。我们从意向性治疗和按方案分析中得出了治疗后表现出持续病毒性应答的患者的总比例。此外，我们还使用现有数据的随机效果模型 (DerSimonian–Laird) 计算了品牌药物和通用直接药物的持续病毒性应答的总相对风险 (RR)。通过 *I^2^* 统计对研究间异质性进行了评估。

**结果:**

我们确定了 19 份研究，涉及来自八个国家或地区的 57 433 名个体。显示持续病毒性应答的患者的总体比例为：按协议分析：98%（95% 置信区间，CI：97–99；18 份研究：*I^2^* = 94.1%）；意向性治疗分析：96%（95% CI：93–98；8 份研究： *I^2^* = 68.1%）。使用品牌药物的持续病毒性应答的可能性与使用通用直接药物的类似 (RR: 1.00; 95% CI: 0.98–1.02; *I^2^* = 0.0%)。与肝硬化患者相比，没有肝硬化的患者的持续病毒性应答的可能性要高很多（RR:1.03；95% CI: 1.01–1.06；7 份研究），但是先前治疗（3 份研究）或人类免疫缺陷病毒合并感染（3 份研究）不会对可能性造成很大的影响。

**结论:**

通用直接药物对丙型肝炎的治疗有显著效果，应考虑在资源有限地区使用，以减轻 HCV 感染患者患肝脏疾病的负担。****

## Резюме

**Цель:**

Сравнение эффективности непатентованных лекарственных препаратов прямого действия и зарегистрированных патентованных лекарственных средств для лечения инфекции, вызванной вирусом гепатита С (ВГС), посредством проведения систематического обзора и метаанализа.

**Методы:**

Авторы провели поиск по сетевым базам данных исследований, в которых сообщалось о достижении стойкого вирусологического ответа через 12 недель после окончания лечения ВГС с использованием непатентованных лекарственных препаратов прямого действия. Авторы получили объединенные пропорции прошедших лечение пациентов с устойчивым вирусологическим ответом в результате анализа совокупности всех начавших лечение пациентов и пациентов без отклонений от протокола лечения. Кроме того, на основе имеющихся данных авторы рассчитали объединенный относительный риск (ОР) устойчивого вирусологического ответа при использовании патентованных лекарственных препаратов по сравнению с непатентованными препаратами прямого действия, используя модель случайных эффектов (DerSimonian — Laird). Неоднородность между исследованиями оценивалась с использованием статистики *I^2^*.

**Результаты:**

Авторы обнаружили 19 исследований, в которых приняли участие 57 433 человека из восьми регионов. Общая доля пациентов с устойчивым вирусологическим ответом составила 98% (95%-й доверительный интервал, ДИ: 97–99; 18 исследований; *I^2^* = 94,1%) в анализе совокупности пациентов без нарушения протокола лечения и 96% (95%-й ДИ: 93–98; 8 исследований; *I^2^* = 68,1%) в анализе совокупности пациентов, начавших лечение. Вероятность устойчивого вирусологического ответа при использовании патентованных лекарственных препаратов была аналогична использованию непатентованных препаратов прямого действия (ОР: 1,00; 95%-й ДИ: 0,98–1,02; *I^2^* = 0,0%). Вероятность достижения устойчивого вирусологического ответа была значительно выше у пациентов, не имеющих цирроза печени (ОР: 1,03; 95%-й ДИ: 1,01–1,06; 7 исследований), но не имела значительной зависимости от предыдущего лечения (3 исследования) или одновременного инфицирования вирусом иммунодефицита человека (3 исследования).

**Вывод:**

Непатентованные лекарственные препараты прямого действия демонстрируют высокую эффективность в лечении гепатита С. Следует рассматривать возможность использования непатентованных препаратов в условиях ограниченных ресурсов для снижения бремени болезней печени у пациентов, инфицированных ВГС.

## Introduction

An estimated 70 million people worldwide are chronically infected by the hepatitis C virus (HCV).^1^ The clinical presentation of HCV infection can vary from minimal fibrosis to cirrhosis and its complications.^2^ The disease is one of the most frequent reasons for liver transplantation and more than 1 million deaths were due to HCV infection in 2013,^3^ most of which were related to cirrhosis and hepatocellular carcinoma.^4^ A sustained virological response to treatment has been associated with lower rates of liver-related complications,^5^ better quality of life,^6^ and a shorter waiting list for liver transplantation among patients with chronic hepatitis C.^7^

The introduction of direct-acting antiviral agents has revolutionized the treatment of chronic hepatitis C – all-oral, interferon-free regimens have been shown to be highly effective.^8^ In 2016, the World Health Organization (WHO) outlined strategies for eliminating HCV infection and for reducing the number of viral hepatitis-related deaths by 65% by 2030.^4^ However, the use of direct-acting agents has had a substantial economic impact in several countries due to high drug costs. Nevertheless, the adoption of a test-and-treat-all strategy is cost–effective and has been shown to be essential for reaching global treatment goals.^9^ Access to direct-acting agents varies widely across the world.^10^ Several countries have provided access with minimal co-payments or have negotiated large discounts with the pharmaceutical industry to provide universal treatment for everyone living with HCV.^11^ Despite the availability of highly effective therapeutic regimens, however, WHO’s target of eliminating HCV infection by 2030 will probably be difficult to achieve for several reasons, including: (i) the high rate of new infections; (ii) HCV-infected individuals remaining untreated due to a lack of screening; (iii) patent restrictions that affect generic medicines; and (iv) the high price of direct-acting agents in middle-income countries with large HCV epidemics.^12^ Generic versions of direct-acting agents could be provided at a much lower cost than brand-name medicines and could contribute to eradicating HCV infection in coming years. Optimally, generic HCV direct-acting agents should be prequalified by WHO.^13^

Our hypothesis was that generic direct-acting agents are highly effective for the treatment of HCV infection. Although observational studies have reported on the effectiveness and safety of generic direct-acting agents in recent years, pooled effectiveness data from published studies is lacking. In this analysis, we estimated the pooled proportions of patients treated with generic direct-acting agents who had a sustained virological response, both with and without comparison with brand-name medicines. 

## Methods

We performed a systemic search of the PubMed®, Embase®, Scopus and LILACS (*Literatura Latino Americana em Ciências da Saúde*) databases to 31 August 2018, without language restrictions. The search string was: [“sofosbuvir” OR “sovaldi” OR “simeprevir” OR “olysio” OR “daclatasvir” OR “daklinza” OR “ledipasvir” OR “harvoni” OR “elbasvir” OR “grazoprevir” OR “zepatier” OR “velpatasvir” OR “epclusa” OR “direct-acting agents”] AND [“hepatitis C” OR “HCV”] AND [“Generic” OR “Drug substitution” OR “Therapeutic equivalency”]. Table 1, Table 2 and Box 1 describe the study inclusion and exclusion criteria. The search strategy is described in detail in the data repository.^14^ Briefly, we searched for randomized or open-label clinical trials or real-life cohort studies that evaluated the effectiveness of generic direct-acting agents in people chronically infected by HCV, with or without comparison with brand-name medicines. In addition, we manually searched the reference lists of included articles and relevant systematic reviews. This systematic review and meta-analysis was registered on PROSPERO (CRD42019117610).^15^

**Table 1:** Study inclusion criteria, systematic review and meta-analysis of generic direct-acting agents for treating hepatitis C

Characteristic	Inclusion criterion	Notes
**Study population**	People living with a chronic HCV infection	None
**Study intervention**	Treatment of HCV infection using generic direct-acting agents	Table 2 lists eligible drugs and their licensed doses and Box 1 lists eligible treatment regimens
**Comparison treatment**	Either: (i) brand-name direct-acting agents for HCV infection; or (ii) no comparator treatment	The following study types were excluded: (i) studies of HCV prevalence or screening; and (ii) clinical trials or cohort studies that evaluated the effectiveness of brand-name direct-acting agents only
**Study outcome**	Sustained virological response 12 weeks after the end of treatment	The outcome used in intention-to-treat and per-protocol analyses was the eradication of HCV virus, as indicated by a sustained virological response 12 weeks after the end of treatment
**Study design**	Randomized or open-label clinical trials and real-life cohort studies	The following study types were eligible for inclusion: (i) randomized or open label clinical trials that compared the effectiveness of generic and brand-name direct-acting agents for the treatment of HCV infection; and (ii) cohort studies that reported the effectiveness of generic direct-acting agents for HCV eradication

HCV: hepatitis C virus.

**Table 2:** Eligible drugs, systematic review and meta-analysis of generic direct-acting agents for treating hepatitis C

Drug	Formulation	Brand name
Sofosbuvir	Tablets containing 400 mg	Sovaldi®
Simeprevir	Capsules containing 150 mg	Olysio®
Daclatasvir	Tablets containing 30 or 60 mg	Daklinza®
Sofosbuvir–ledipasvir combination	Tablets containing 400 mg of sofosbuvir and 90 mg of ledipasvir	Harvoni®
Sofosbuvir–velpatasvir combination	Tablets containing 400 mg of sofosbuvir and 100 mg of velpatasvir	Epclusa®
Grazoprevir–elbasvir combination	Tablets containing 100 mg of grazoprevir and 50 mg of elbasvir	Zepatier®

Box 1 **Eligible drug treatment regimes, systematic review and meta-analysis of generic direct-acting agents for treating hepatitis C, 2019**

• Sofosbuvir and daclatasvir, with or without ribavirin for 12 or 24 weeks.

• Sofosbuvir and simeprevir, with or without ribavirin for 12 or 24 weeks.

• Sofosbuvir–daclatasvir combination, with or without ribavirin for 12 or 24 weeks.

• Sofosbuvir–ledipasvir combination, with or without ribavirin for 8 or 12 weeks.

• Sofosbuvir–velpatasvir combination, with or without ribavirin for 12 weeks.

• Grazoprevir–elbasvir combination, with or without ribavirin for 12 weeks.

Two independent reviewers screened the titles and abstracts of all articles identified for eligibility using the Rayyan QRCI web application and a list of inclusion and exclusion criteria.^16^ A response to treatment was defined as a sustained virological response 12 weeks after the end of treatment. We excluded conference papers, editorials, published letters, studies in children or adolescents younger than 18 years, studies that exclusively evaluated the effectiveness of brand-name direct-acting agents and studies that did not report sustained virological response data.

Two investigators extracted the following data from the full text of each included study and entered them in a case report form using the database application REDCap (Research Electronic Data Capture):^17^ study design, study country, period of recruitment, participants’ demographic characteristics, direct-acting agent regimens used, duration of direct-acting agent treatment, previous treatment, presence of cirrhosis, presence of human immunodeficiency virus (HIV) coinfection, country of manufacture of generic direct-acting agents, trade names of generic direct-acting agents and the proportions of patients with sustained virological response from per-protocol or intention-to-treat analyses or both. This systematic review and meta-analysis was performed in accordance with the Preferred Reporting Items for Systematic reviews and Meta-Analyses statement.^18^

The quality of the studies included was appraised using the National Institute of Health’s quality assessment tool for observational cohort and cross-sectional studies.^19^ This tool’s 14-item checklist was designed to focus on factors important for evaluating a study’s internal validity. Studies were rated as being of good, fair or poor quality. Those with 0 to 6, 7 to 10, or 11 or more “yes” responses to the 14 items were considered as having a high, moderate or low risk of bias, respectively.

### Statistical analysis

Our primary outcome was the pooled proportions of treated patients with sustained virological response for generic direct-acting agents, reported with a 95% confidence interval (CI). In addition, where data were available, we performed a meta-analysis of proportions using a random-effects model (i.e. the DerSimonian–Laird method) to calculate the pooled relative risk (RR) of a sustained virological response with brand-name compared with generic direct-acting agents. Between-study heterogeneity was assessed using the *I^2^* statistic: an *I^2^* value of 25– < 50%, 50–75%, and > 75% was considered to indicate mild, moderate or severe heterogeneity, respectively.^20^ We performed subgroup analyses to explore how the following variables affected the pooled proportions of sustained virological response and heterogeneity: (i) the presence of cirrhosis; (ii) previous treatment; and (iii) the presence of an HIV–HCV coinfection. In addition, we performed sensitivity analyses to evaluate the impact of the study’s geographical location and quality on the sustained virological response proportions and heterogeneity. A *P*-value ≤ 0.05 was regarded as significant. All statistical analyses were conducted using the metan and metaprop procedures in Stata v.14 (StataCorp LP., College Station, United States of America).^21^^,^^22^

## Results

### Study characteristics

**Fig. 1:**
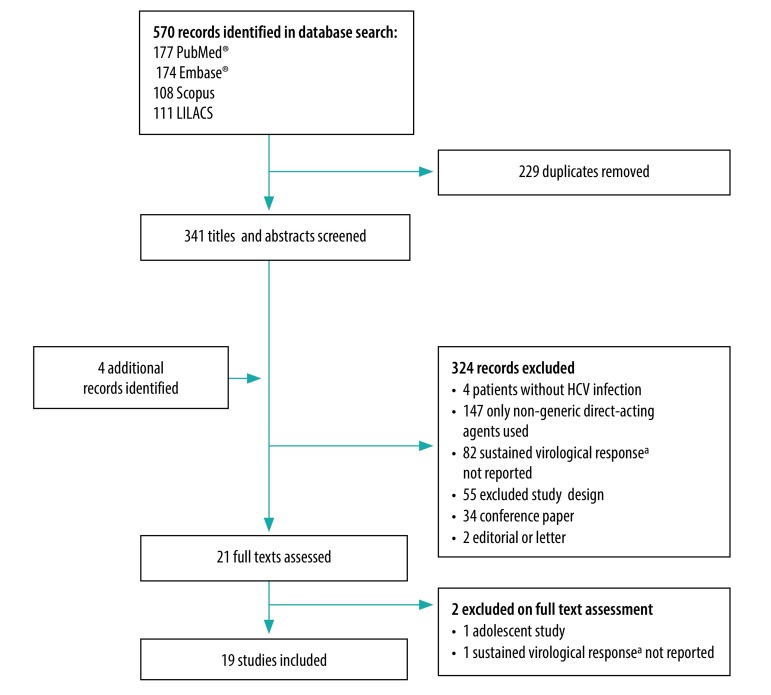
Study selection flowchart, systematic review and meta-analysis of generic direct-acting agents for treating hepatitis C

The database and manual searches identified 341 and 4 records, respectively. Subsequent screening of titles and abstracts led to 19 studies being eligible for inclusion in the meta-analysis (Fig. 1).^23^^–^^41^ These 19 published full articles reported sustained virological response proportions for generic direct-acting agents in a total of 57 433 individuals and all except one were published in English.^38^ The studies were performed in seven territories – Egypt (seven studies), India (three studies), China (four studies), the Islamic Republic of Iran (two studies), Argentina (one study) and Chile (one study) – and one was a multiregional study in Australia, eastern Europe and South-East Asia (Table 3; available at: http://www.who.int/bulletin/volumes/98/3/19-231522). Four studies compared the effectiveness of generic and brand-name direct-acting agents.^23^^,^^24^^,^^32^^,^^38^ Patients were treated with generic versions of: (i) sofosbuvir and ribavirin; (ii) sofosbuvir and daclatasvir, with or without ribavirin; (iii) sofosbuvir and ledipasvir, with or without ribavirin; or (iv) sofosbuvir and velpatasvir. Cirrhosis was identified by liver biopsy, liver stiffness measurement, serological biomarkers, clinical signs or imaging. Generic direct-acting agents originated from Egypt (nine studies), India (seven studies), the Islamic Republic of Iran (two studies), Argentina (one study) and Bangladesh (two studies), though one study had multiregional sources (Table 4; available at: http://www.who.int/bulletin/volumes/98/3/19-231522). Study quality was good in 37% (7/19), fair in 26% (5/19) and poor in 37% (7/19) and the risk of bias was low in 37% (7/19), moderate in 52% (10/19) and high in 11% (2/19). Three studies used WHO prequalified medicines or medicines listed for use in mass-treatment programmes by the Expert Review Panel of the Global Fund to Fight AIDS, Tuberculosis and Malaria (Table 4).^43^ In addition, another three studies used generic direct-acting agents whose bioequivalence with the original versions had previously been demonstrated in pharmacokinetics studies.

HCV: hepatitis C virus.

^a^ A sustained virological response 12 weeks after the end of treatment.

**Table 3:** Characteristics of included studies in the systematic review and meta-analysis of generic direct-acting agents for treating hepatitis C, 2016–2018

Study	Location	Multicentre study	Study period	Comparison with brand-name direct-acting agent	Generic direct-acting agent treatment regimen	Treatment duration, weeks	Method of cirrhosis diagnosis	No. of patients	No. (%) of patients with specific HCV genotypes^a^	No. (%) of male patients	No. (%) of previously treated patients	No. (%) of patients with cirrhosis	No. (%) patients with an HIV coinfection
Yakoot et al., 2016^39^	Egypt	Yes	ND	No	SOF and RBV	12 or 24	FIB-4 or APRI	50	genotype 4: 50 (100)	26 (52)	12 (24)	11 (22)	0 (0)
Hill et al., 2017^27^	Multiregional (Australia, Eastern Europe and South-East Asia)	Yes	ND	No	(i) SOF and DCV; and (ii) SOF–LDV combination	ND	ND	250	ND	ND	ND	ND	ND
Merat et al., 2017^33^	Iran (Islamic Republic of)	No	Sep 2015 to Nov 2015	No	SOF–DCV combination and RBV	12	Liver biopsy, liver stiffness measurement, clinical signs or imaging	100	genotype 1: 56 (56); genotype 3: 44 (44)	65 (65)	ND	100 (100)	0 (0)
Nagral et al., 2017^34^	India	Yes	ND	No	(i) SOF and DCV ± RBV; and (ii) SOF–LDV combination ± RBV	12 or 24	Liver stiffness measurement, clinical signs or imaging	29	genotype 1: 17 (59); genotype 3: 12 (41)	16 (55)	7 (24)	6 (21)	0 (0)
Sharafi et al., 2017^36^	Iran (Islamic Republic of)	No	ND	No	SOF–LDV combination ± RBV	12 or 24	Liver stiffness measurement, clinical signs or imaging	30	genotype 1: 29 (97); genotype 4: 1 (3)	22 (73)	18 (60)	16 (53)	0 (0)
Vargas et al., 2017^38^	Chile	Yes	Jun 2013 to May 2017	Yes	(i) SOF and DCV ± RBV; and (ii) SOF–LDV combination ± RBV	ND	Liver biopsy, liver stiffness measurement, clinical signs or imaging	76	ND	ND	ND	ND	ND
Yakoot et al., 2017^40^	Egypt	ND	ND	No	SOF and DCV	8 or 12	Liver stiffness measurement, FIB-4 or APRI	120	genotype 4: 120 (100)	48 (40)	29 (24)	0 (0)	0 (0)
Zeng et al., 2017^41^	China	ND	ND	No	SOF–LDV combination ± RBV	8 or 12	Liver stiffness measurement, clinical signs or imaging	192	genotype 1: 192 (100)	38 (20)	ND	63 (33)	0 (0)
Abozeid et al., 2018^23^	Egypt	No	Jan 2016 to Dec 2017	Yes	(i) SOF and DCV ± RBV; and (ii) SOF–LDV combination ± RBV	12 or 24	Liver biopsy, liver stiffness measurement, FIB-4, APRI, clinical signs or imaging	395	ND	226 (57)	27 (7)	148 (37)	ND
El-Nahaas et al., 2018^24^	Egypt	No	ND	Yes	SOF and DCV ± RBV	12	FIB-4 or APRI	234	ND	139 (59)	50 (21)	61 (26)	0 (0)
Elsharkawy et al., 2018^25^	Egypt	Yes	Oct 2015 to Mar 2016	No	SOF and DCV ± RBV	12	ND	36 186	ND	ND	ND	ND	ND
Gupta et al., 2018^26^	India	No	May 2015 to Jan 2017	No	(i) SOF and RBV; (ii) SOF and DCV ± RBV; and (iii) SOF–LDV combination ± RBV	12 or 24	Liver biopsy, liver stiffness measurement, clinical signs or imaging	393	genotype 1: 83 (21); genotype 3: 310 (79)	ND	ND	ND	0 (0)
Kumar et al., 2018^28^	India	ND	Sep 2015 to Feb 2017	No	(i) SOF and RBV; (ii) SOF and DCV; and (iii) SOF–LDV combination	12 or 24	Liver biopsy, clinical signs or imaging	71	genotype 1: 44 (62); genotype 3: 27 (38)	54 (76)	13 (18)	17 (24)	ND
Liu et al., 2018^31^	Taiwan, China	No	Aug 2016 to Apr 2017	No	SOF–VEL combination ± RBV	12	Liver stiffness measurement	228	genotype 1: 113 (50); genotype 2: 89 (39); genotype 3: 7 (3); genotype 4: 3 (1)	137 (60)	58 (25)	52 (23)	69 (30)
Liu et al., 2018^30^	Taiwan, China	Yes	May 2016 to Jun 2017	No	(i) SOF and RBV; (ii) SOF–DCV combination ± RBV; (iii) SOF–LDV combination ± RBV; and (iv) SOF–VEL combination ± RBV	12 or 24	Liver biopsy, liver stiffness measurement, FIB-4, APRI, clinical signs or imaging	517	genotype 1: 297 (57); genotype 2: 185 (36); genotype 3: 8 (2); genotype 4: 2 (1)	252 (49)	147 (28)	187 (36)	61 (12)
Li et al., 2018^29^	China	Yes	Jun 2015 to Dec 2016	No	(i) SOF and RBV; (ii) SOF and DCV ± RBV; and (iii) SOF–LDV combination ± RBV	12 or 24	Clinical signs or imaging	137	genotype 1: 44 (32); genotype 2: 3 (2); genotype 3: 71 (52)	110 (80)	ND	26 (19)	137 (100)
Marciano et al., 2018^32^	Argentina	Yes	Mar 2016 to Jun 2016	Yes	(i) SOF and RBV; and (ii) SOF and DCV ± RBV	12 or 24	Liver biopsy, liver stiffness measurement, clinical signs or imaging	321	genotype 1: 240 (75); genotype 2: 27 (8); genotype 3: 47 (15); genotype 4: 7 (2)	189 (59)	136 (42)	292 (91)	58 (18)
Omar et al., 2018^35^	Egypt	Yes	Nov 2015 to Dec 2015	No	SOF and DCV ± RBV	12	Liver stiffness measurement or FIB-4	18 378	ND	7798 (42)	1296 (7)	ND	ND
Shousha et al., 2018^37^	Egypt	ND	Feb 2017 to Jul 2017	No	SOF–LDV combination ± RBV	8 or 12	Liver stiffness measurement	40	genotype 4: 40 (100)	17 (43)	ND	0 (0)	0 (0)

APRI: aspartate aminotransferase-to-platelet ratio index; DCV: daclatasvir; FIB-4: fibrosis-4 score; HCV: hepatitis C virus; HIV: human immunodeficiency virus; LDV: ledipasvir; ND: not determined; RBV: ribavirin; SOF: sofosbuvir; VEL: velpatasvir.

^a^ The number of patients with specific HCV genotypes does not always equal the total number of patients because data on HCV genotype were missing for some patients in a few studies.

**Table 4:** Generic medicines used, systematic review and meta-analysis of generic direct-acting agents for treating hepatitis C, 2019

Study and generic direct-acting agents used	Commercial name	Manufacturer	Quality assessment		
			WHO prequalification	Listed by the Global Fund’s Expert Review Panel	Other
**Yakoot et al., 2016**^39^					
SOF (400 mg)	Gratisovir®	Pharco Pharmaceutical (Egypt)	No	No	No
SOF (400 mg)	Grateziano®	European Egyptian Pharmaceutical Industries (Egypt)	Yes (reference: HP003)	No	No
**Hill et al., 2017**^27^					
SOF (400 mg), DCV (60 mg), LDV (90 mg)	Numerous	Direct-acting agents from 24 different companies; 34% from Cipla Ltd (Egypt) and 30% from Hetero Laboratory Ltd (India)	Yes (SOF from Cipla Ltd and Hetero Laboratory Ltd)	Yes (DCV from Cipla Ltd and Hetero Laboratory Ltd)	No
**Merat et al., 2017**^33^					
SOF–DCV combination (400/60 mg)	Sovodak®	Fanavaran Rojan Mohaghegh Darou (Islamic Republic of Iran)	No	No	No
**Nagral et al., 2017**^34^					
SOF (400 mg), DCV (60 mg), SOF–LDV combination (400/90 mg)	Not reported	All direct-acting agents manufactured in India	ND	ND	ND
**Sharafi et al., 2017**^36^					
SOF–LDV combination (400/90 mg)	Sobopasvir®	Sobhan Medicine Trade Development Co. (Islamic Republic of Iran)	No	No	No
**Vargas et al., 2017**^38^					
SOF (400 mg), DCV (60 mg), SOF–LDV combination (400/90 mg)	Not reported	Most direct-acting agents manufactured in India	ND	ND	ND
**Yakoot et al., 2017**^40^					
SOF (400 mg)	Gratisovir®	Pharco Pharmaceutical (Egypt)	No	No	No
DCV (60 mg)	Daktavira®	European Egyptian Pharmaceutical Industries (Egypt)	No	No	No
**Zeng et al., 2017**^41^					
SOF–LDV combination (400/90 mg)	Hepcinat LP®	Natco Pharma (India)	No	No	No
**Abozeid et al., 2018**^23^					
SOF (400 mg)	Gratisovir®	Pharco Pharmaceutical (Egypt)	No	No	No
DCV (60 mg)	Daktavira®	European Egyptian Pharmaceutical Industries (Egypt)	No	No	No
SOF–LDV combination (400/90 mg)	MPI-Viropack-Plus®	Marcyrl Pharmaceutical Industries (Egypt)	No	No	Bioequivalence shown for SOF–LDV combination versus Harvoni®^42^
**El-Nahaas et al., 2018**^24^					
SOF (400 mg)	Sofolanork®	Mash Premiere (Egypt)	No	No	No
DCV (60 mg)	Daklanork®	Mash Premiere (Egypt)	No	No	No
**Elsharkawy et al., 2018**^25^					
SOF (400 mg), DCV (60 mg)	Not reported	All direct-acting agent s manufactured in Egypt	ND	ND	ND
**Gupta et al., 2018**^26^					
SOF (400 mg)	Hepcvir®	Cipla Ltd (Egypt)	Yes (reference: HP004)	ND	ND
DCV (60 mg)	Hepdac®	Cipla Ltd (Egypt)^a^	Yes (reference: HP008)	ND	ND
SOF–LDV combination (400/90 mg)	Not reported	The direct-acting agent combination was manufactured in India^b^	No	No	No
**Kumar et al., 2018**^28^					
SOF (400 mg), DCV (60 mg), SOF–LDV combination (400/90 mg)	Not reported	All direct-acting agents manufactured in India	ND	ND	ND
**Liu et al., 2018**^31^					
SOF–VEL combination (400/100 mg)	Sofosvel®	Beacon Pharmaceuticals (Bangladesh)	No	No	No
**Liu et al., 2018**^30^					
SOF (400 mg)	Hepcinat®	Natco Pharma (India)	No	No	Bioequivalence shown for SOF versus Sovaldi®^13^
SOF–DCV combination (400/60 mg)	Darvoni®	Beacon Pharmaceuticals (Bangladesh)	No	No	No
SOF–LDV combination (400/90 mg)	Hepcinat-LP®	Natco Pharma (India)	No	No	No
SOF–LDV combination (400/90 mg)	Ledifos®	Hetero Laboratory Ltd (India)	No	No	No
SOF–VEL combination (400/100 mg)	Velpanat®	Natco Pharma (India)	No	No	No
SOF–VEL combination (400/100 mg)	Velasof®	Hetero Laboratories Ltd (India)	No	No	No
**Li et al., 2018**^29^					
SOF (400 mg), DCV (60 mg), SOF–LDV combination (400/90 mg)	Not reported	All direct-acting agents manufactured in India	ND	ND	ND
**Marciano et al., 2018**^32^**^,c^**					
SOF (400 mg)	Probirase®	Laboratorios Richmond SACIF (Argentina)	No	No	No
**Omar et al., 2018**^35^					
SOF (400 mg), DCV (60 mg)	Numerous	AUG Pharma, Magic Pharma, Marcyrl Pharmaceutical Industries and Pharco Pharmaceutical (all Egypt)	No	No	Bioequivalence shown for SOF versus Sovaldi® and for DCV versus Daklinza® (Marcyrl Pharmaceutical Industries)^42^
**Shousha et al., 2018**^37^					
SOF–LDV combination (400/90 mg)	MPI-Viropack Plus®	Marcyrl Pharmaceutical Industries (Egypt)	No	No	Bioequivalence shown for SOF–LDV combination versus Harvoni®^42^

DCV: daclatasvir; Global Fund: Global fund to Fight AIDS, Tuberculosis and Malaria; LDV: ledipasvir; NA: not applicable; ND: not determined; SOF: sofosbuvir; VEL: velpatasvir.

^a^ The generic drug was produced by Cipla Ltd in collaboration with the Bristol-Myers Squibb Co. through the Medicines Patent Pool.

^b^ The SOF–LDV combination was produced by Indian companies using voluntary manufacturing licences from Gilead Sciences Inc.

^c^ In this study, patients received generic sofosbuvir (Probirase®) and brand-name daclatasvir (Daklinza®) from the Bristol-Myers Squibb Co.

### Sustained virological response

#### Overall

**Fig. 2:**
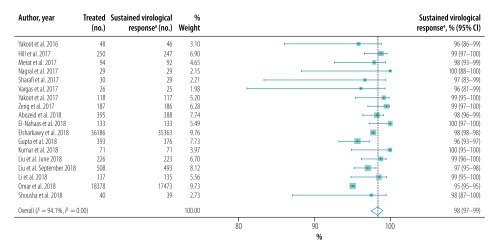
Sustained virological response to hepatitis C treatment by generic direct-acting agents, per-protocol analysis, systematic review and meta-analysis, 2019

**Fig. 3:**
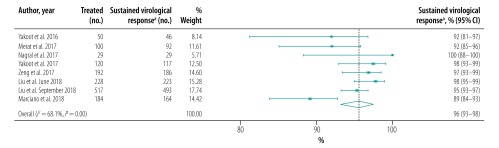
Sustained virological response to hepatitis C treatment by generic direct-acting agents, intention-to-treat analysis, systematic review and meta-analysis, 2019

**Fig. 4:**
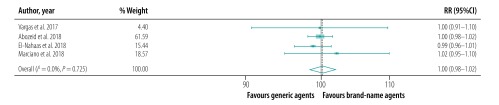
Relative risk of a sustained virological response to hepatitis C treatment by brand-name versus generic direct-acting agents, systematic review and meta-analysis, 2019

CI: confidence interval; RR: relative risk.

**Fig. 5:**
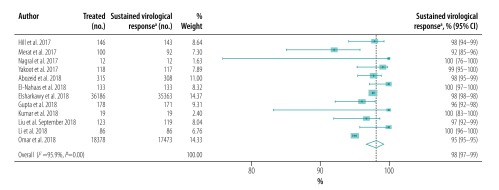
Sustained virological response to hepatitis C treatment with generic sofosbuvir and daclatasvir, with or without ribavirin, systematic review and meta-analysis, 2019

**Fig. 6:**
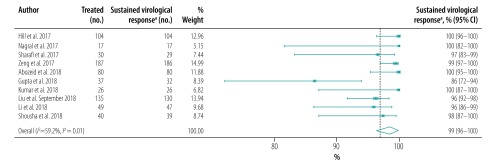
Sustained virological response to hepatitis C treatment with generic sofosbuvir and ledipasvir, with or without ribavirin, systematic review and meta-analysis, 2019

The pooled proportion of patients with sustained virological response for generic direct-acting agents overall was 98% (95% CI: 97–99; *I^2^* = 94.1%) in per-protocol analyses (18 studies including 57 249 patients; Fig. 2) and 96% (95% CI: 93–98; *I^2^* = 68.1%) in intention-to-treat analyses (8 studies including 1420 patients; Fig. 3). The likelihood of a sustained virological response with brand-name medicines was similar to that with generic direct-acting agents (RR: 1.00; 95% CI: 0.98–1.02; *I^2^* = 0.0%) in the four studies (including 1026 patients) that compared the two types of direct-acting agent (Fig. 4). Among the 55 788 patients treated with sofosbuvir and daclatasvir, with or without  ribavirin, the pooled proportion was 98% (95% CI: 97–99; *I^2^* = 96.1%) in per-protocol analyses (Fig. 5; available at: http://www.who.int/bulletin/volumes/98/3/19-231522). Among the 705 treated by sofosbuvir and ledipasvir, with or without  ribavirin, the pooled proportion was 99% (95% CI: 96–100; *I^2^* = 59.2%) in per-protocol analyses (Fig. 6; available at: http://www.who.int/bulletin/volumes/98/3/19-231522). We could not calculate pooled proportion for patients treated with sofosbuvir and ribavirin or sofosbuvir and velpatasvir because there were too few studies or participants. In the two studies in which HCV monoinfected patients received the generic version of the pan-genotypic regimen of sofosbuvir and velpatasvir, the proportion were 98% (95% CI: 95–99) and 99% (95% CI: 97–100), respectively.

CI: confidence interval.

^a^ A sustained virological response 12 weeks after the end of treatment.

CI: confidence interval.

^a^ A sustained virological response 12 weeks after the end of treatment.

CI: confidence interval.

^a^ A sustained virological response 12 weeks after the end of treatment.

CI: confidence interval.

^a^ A sustained virological response 12 weeks after the end of treatment.

#### Subgroups

**Fig. 7:**
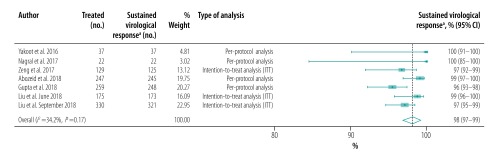
Sustained virological response in patients without cirrhosis to hepatitis C treatment with generic direct-acting agents, systematic review and meta-analysis, 2019

**Fig. 8:**
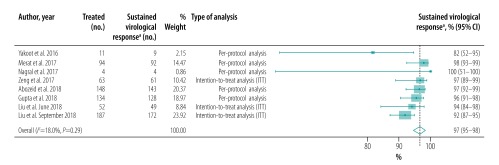
Sustained virological response in patients with cirrhosis to hepatitis C treatment with generic direct-acting agents, systematic review and meta-analysis, 2019

**Fig. 9:**
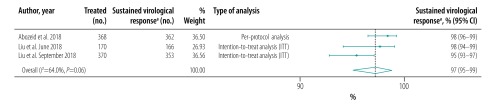
Sustained virological response in treatment-naïve patients to hepatitis C treatment with generic direct-acting agents, systematic review and meta-analysis, 2019

**Fig. 10:**
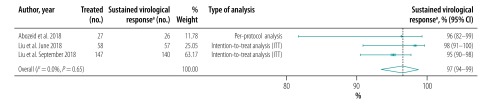
Sustained virological response in previously treated patients to hepatitis C treatment with generic direct-acting agents, systematic review and meta-analysis, 2019

**Fig. 11:**
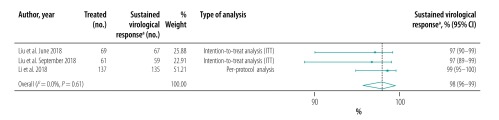
Sustained virological response in patients with an HIV coinfection to hepatitis C treatment with generic direct-acting agents, systematic review and meta-analysis, 2019

CI: confidence interval; HIV: human immunodeficiency virus.

^a^ A sustained virological response 12 weeks after the end of treatment.

A single study exclusively included individuals with cirrhosis,^33^ 11 studies included patients with and without cirrhosis, two excluded cirrhotic patients and five did not report the prevalence of cirrhosis. Of the eight studies that reported proportions of sustained virological response in patients with cirrhosis, seven reported proportions for cirrhotic and noncirrhotic patients separately.^23^^,^^26^^,^^30^^,^^31^^,^^34^^,^^39^^,^^41^ The pooled proportion for patients without and with cirrhosis was 98% (95% CI: 97–99; *I^2^* = 34.2%; 7 studies; 1199 patients; Fig. 7; available at: http://www.who.int/bulletin/volumes/98/3/19-231522) and 97% (95% CI: 95–98; *I^2^* = 18.0%; 8 studies; 693 patients; Fig. 8; available at: http://www.who.int/bulletin/volumes/98/3/19-231522), respectively. The likelihood of a sustained virological response was significantly higher in patients without cirrhosis than in those with the disease (RR: 1.03; 95% CI: 1.01–1.06) in the seven studies that included both cirrhotic and noncirrhotic individuals (Table 5). Only three studies reported proportions of sustained virological response in treatment-naïve and previously treated HCV-infected patients (Table 6; available at: http://www.who.int/bulletin/volumes/98/3/19-231522).^23^^,^^30^^,^^31^ The pooled proportion was 97% (95% CI: 95–99; *I^2^* = 64.0%; 908 patients; Fig. 9; available at: http://www.who.int/bulletin/volumes/98/3/19-231522) in treatment-naïve patients and 97% (95% CI: 94–99; *I^2^* = 0.0%; 232 patients; Fig. 10; available at: http://www.who.int/bulletin/volumes/98/3/19-231522) in previously treated patients. Previous treatment had no significant effect on the likelihood of a sustained virological response (RR: 1.00; 95% CI: 0.97–1.03). The presence of an HIV coinfection was an exclusion criterion in nine studies and four did not report the proportion of patients with an HIV coinfection. Only one study included exclusively HIV–HCV coinfected patients,^29^ whereas two other studies reported sustained virological responses in HIV–HCV coinfected and HCV monoinfected patients (Table 7; available at: http://www.who.int/bulletin/volumes/98/3/19-231522).^30^^,^^31^ The pooled proportion in HIV–HCV coinfected patients was 98% (95% CI: 96–99; *I^2^* = 0.0%; 3 studies; 267 patients; Fig. 11; available at: http://www.who.int/bulletin/volumes/98/3/19-231522). There was no significant difference in the likelihood of a sustained virological response between HIV–HCV coinfected and HCV monoinfected patients (RR: 1.00; 95% CI: 0.96–1.03).

CI: confidence interval.

^a^ A sustained virological response 12 weeks after the end of treatment.

CI: confidence interval.

^a^ A sustained virological response 12 weeks after the end of treatment.

**Table 5:** Effect of cirrhosis on the likelihood of a sustained virological response^a^ to generic direct-acting agents in patients with hepatitis C, meta-analysis, 2019

Study^b^	No. of patients with a response/no. treated		RR (95% CI)	Study weighting (%)
	Without cirrhosis	With cirrhosis		
Yakoot et al., 2016^39^	37/37	9/11	1.19 (0.90–1.58)	1.87
Nagral et al., 2017^34^	22/22	3/4	1.20 (0.77–1.87)	0.88
Zeng et al., 2017^41^	125/129	61/63	1.00 (0.95–1.06)	11.00
Abozeid et al., 2018^23^	245/247	143/148	1.03 (0.99–1.06)	24.00
Gupta et al., 2018^26^	248/259	128/134	1.00 (0.96–1.05)	22.64
Liu et al., 2018^31^	173/175	49/52	1.05 (0.98–1.12)	10.14
Liu et al., 2018)^30^	330/321	172/187	1.06 (1.01–1.11)	29.47
**Pooled data^c^**	**1180/1190**	**565/599**	**1.03 (1.01–1.06)**	**100.00**

CI: confidence interval; RR: relative risk.

^a^ A response was defined as a sustained virological response 12 weeks after the end of treatment.

^b^ The Merat et al.^33^ study was not included in this subanalysis because it involved only patients with cirrhosis.

^c^ The *I^2^* value for between-study heterogeneity was 0.0% (*P* = 0.435).

**Table 6:** Effect of previous treatment on the likelihood of a sustained virological response^a^ to generic direct-acting agents in patients with hepatitis C, meta-analysis, 2019

Study	No. of patients with a response/no. treated		RR (95% CI)	Study weighting (%)
	Treatment-naïve	Previously treated		
Abozeid et al., 2018^23^	362/368	26/27	1.02 (0.95–1.10)	14.51
Liu et al., 2018^31^	166/170	57/58	0.99 (0.95–1.04)	25.46
Liu et al., 2018^30^	353/370	140/147	1.00 (0.96–1.06)	60.03
**Pooled data^b^**	**881/908**	**223/232**	**1.00 (0.97–1.03)**	**100.00**

CI: confidence interval; RR: relative risk.

^a^ A response was defined as a sustained virological response 12 weeks after the end of treatment.

^b^ The *I^2^* value for between-study heterogeneity was 0.0% (*P* = 0.810).

CI: confidence interval.

^a^ A sustained virological response 12 weeks after the end of treatment.

CI: confidence interval.

^a^ A sustained virological response 12 weeks after the end of treatment.

**Table 7:** Effect of an HIV coinfection on the likelihood of a sustained virological response^a^ to generic direct-acting agents in patients with hepatitis C, meta-analysis, 2019

Study^b^	No. of patients with a response/no. treated		RR (95% CI)	Study weighting (%)
	With an HCV monoinfection	With an HIV–HCV coinfection		
Liu et al., 2018^31^	156/159	67/69	1.01 (0.97–1.06)	47.31
Liu et al., 2018^30^	434/456	59/61	0.98 (0.94–1.04)	52.69
**Pooled data^c^**	**590/615**	**126/130**	**1.00 (0.96–1.03)**	**100.00**

CI: confidence interval; HCV: hepatitis C virus; HIV: human immunodeficiency virus; RR: relative risk.

^a^ A response was defined as a sustained virological response 12 weeks after the end of treatment.

^b^ The Li et al.^29^ study was not included in this subanalysis because it involved only patients with an HIV–HCV coinfection.

^c^ The *I^2^* value for between-study heterogeneity was 0.0% (*P* = 0.842).

## Sensitivity analysis

**Fig. 12:**
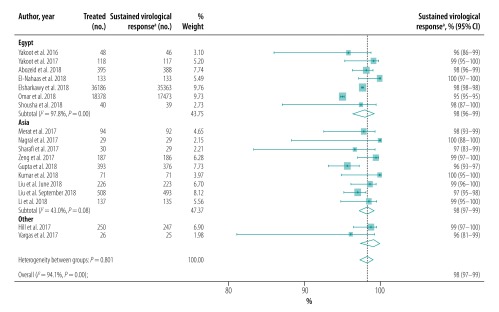
Sustained virological response to hepatitis C treatment with generic direct-acting agents, by geographical location, systematic review and meta-analysis, 2019

**Fig. 13:**
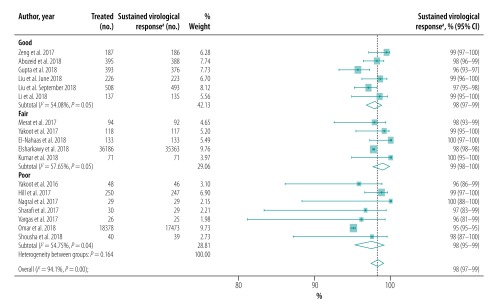
Sustained virological response to hepatitis C treatment with generic direct-acting agents, by study quality, systematic review and meta-analysis, 2019

**Fig. 14:**
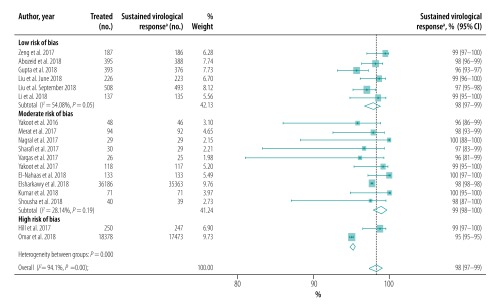
Sustained virological response to hepatitis C treatment with generic direct-acting agents, by risk of study bias, systematic review and meta-analysis, 2019

Our sensitivity analysis showed that heterogeneity was lower in studies performed in Asia than in Egypt (Fig. 12). In addition, we found that heterogeneity was lower in studies of patients with cirrhosis (Fig. 8) and when studies were stratified by quality (Fig. 13; available at: http://www.who.int/bulletin/volumes/98/3/19-231522) or risk of bias (Fig. 14; available at: http://www.who.int/bulletin/volumes/98/3/19-231522).

CI: confidence interval.

^a^ A sustained virological response 12 weeks after the end of treatment.

CI: confidence interval.

^a^ A sustained virological response 12 weeks after the end of treatment.

Notes: The quality of each study was rated as good, fair or poor (see main text for details).

CI: confidence interval.

^a^ A sustained virological response 12 weeks after the end of treatment.

Notes: The risk of bias in each study was rated as low, moderate or high (see main text for details).

## Discussion

Through a systematic review and meta-analysis approach, we derived pooled proportions of sustained virological response in patients treated for HCV infection using generic direct-acting agents. We found that generic direct-acting agents were highly effective. The overall pooled proportion of patients with a sustained virological response was 98% in real-life observational studies that included over 57 000 individuals, which was similar to that reported for brand-name direct-acting agents in large, real-life, observational cohort studies around the world.^8^^,^^44^^,^^45^ In particular, we found that a sustained virological response with generic direct-acting agents was similar to brand-name medicines. Additionally, in sensitivity analyses, we found that sustained virological response was also high with specific regimens, such as sofosbuvir with daclatasvir and sofosbuvir with ledipasvir. Although neither an HIV coinfection nor previous treatment was associated with a high treatment failure, the presence of cirrhosis at baseline was associated with a significantly lower sustained virological response in patients treated with generic direct-acting agents. The results of this study can help in the elaboration of public health strategies for using generic direct-acting agents to treat HCV infection.

Our study findings have implications for achieving the goal of eliminating HCV infection by 2030.^4^ Universal access to direct-acting agents is essential for decreasing viral transmission as well as for reducing mortality and the risk of liver-related complications associated with chronic hepatitis C worldwide. However, HCV treatment has entailed a substantial financial burden, especially as direct-acting agents are expensive.^46^ The nominal price of 12-week course of sofosbuvir ranges from 6 766 United States dollars (US$) in Brazil to US$ 64 680 in the United States.^11^^,^^47^ In contrast, a course of a generic direct-acting agent regimen can be produced for approximately US$ 200 per patient in countries such as Egypt and India.^27^

The production of generic direct-acting agents has been challenged in various local intellectual property jurisdictions because some pharmaceutical components may still be patented. In most countries, local drug regulatory authorities can approve the marketing of a generic version of a patented drug only after the relevant patent has expired, generally after 20 years.^48^ In several countries, local intellectual property offices have evaluated requests to cancel patent claims previously granted to pharmaceutical companies, thereby opening up the possibility that affordable generic versions of direct-acting agents could be produced.^49^ In opposition, pharmaceutical companies have defended their patents and, in the meantime, have collaborated with local companies to produce authorized versions of generic medicines for HCV treatment. The cost of these authorized versions will most likely exceed that of generic direct-acting agents produced by independent companies. Authorized, generic versions of sofosbuvir–ledipasvir and sofosbuvir–velpatasvir combinations were expected to be available in the United States in 2019 at a cost of US$ 24 000 per treatment course.^50^

Our study has limitations. First, there was high between-study heterogeneity for pooled overall proportions of sustained virological response. High heterogeneity might have resulted from differences in the ethnic or clinical characteristics of study participants. Most studies were conducted either in Egypt, where most patients have an HCV genoype-4 infection, or in various parts of Asia. Our sensitivity analysis showed that the region where the study took place and characteristics of patients and study design influenced the heterogeneity. Second, there was a lack of a pooled proportion of patients with a sustained virological response for pan-genotypic interferon-free regimens. We acknowledge that few studies included patients treated with sofosbuvir and velpatasvir or patients with an HIV–HCV coinfection.^30^^,^^31^ Most studies included in our analysis were real-life cohort studies involving a heterogeneous group of HCV-infected patients treated using different generic, interferon-free regimens. Third, there was a low number and quality of studies that compared generic and brand-name direct-acting agents. We did not identify any randomized clinical trials that compared generic and brand-name direct-acting agents. In the four studies included, the choice between brand-name and generic medicines was influenced by local guidelines on the treatment of HCV infection, physicians’ and patients’ preferences, insurance approval and the availability of generic direct-acting agents.^23^^,^^24^^,^^32^^,^^38^ Fourth, the original studies’ used generic medicines that were not prequalified by WHO. Our analysis included only six studies that used medicines that were either prequalified by WHO, listed by the Global Fund’s Expert Review Panel or had been demonstrated to be bioequivalent to a brand-name medicine. This was probably because the studies identified included patients who were treated for an HCV infection between 2015 and 2017, before most generic direct-acting agents had been prequalified by WHO. It is important, however, that the quality of the generic direct-acting agents used for HCV treatment should have been assessed, particularly through WHO’s prequalification process.

The main strength of our study is the large number of patients in real-life scenarios included in the meta-analysis. This large sample size enabled us to estimate the pooled overall proportion of patients with a sustained virological response rates and proportions for different direct-acting agent regimens and for the presence of conditions such as cirrhosis. Moreover, we were able to perform sensitivity analyses that explored the effect on pooled estimates of geographical location, study quality and clinical and demographic characteristics.

In conclusion, we found that the proportion of patients treated with generic direct-acting agents with a sustained virological response was high. The proportion was also high in patients treated with sofosbuvir and daclatasvir, and with sofosbuvir and ledipasvir, and in those with cirrhosis or an HIV coinfection. Recent cost–effectiveness studies of generic direct-acting agents in India suggest that their use can reduce costs,^51^ especially if pan-genotypic regimens are used (though efficacy estimates for brand-name medicines were used in these studies).^52^ Our results corroborate these economic analyses by showing that the effectiveness of generic and brand-name direct-acting agents is indeed the same. Future cost–effectiveness analyses are needed to investigate the specific characteristics of different countries and regions. Nevertheless, generic direct-acting agents are effective and should be considered in public health strategies for HCV elimination.
